# Extensive Dissemination of Methicillin-Resistant *Staphylococcus aureus* (MRSA) between the Hospital and the Community in a Country with a High Prevalence of Nosocomial MRSA

**DOI:** 10.1371/journal.pone.0059960

**Published:** 2013-04-03

**Authors:** Diana Espadinha, Nuno A. Faria, Maria Miragaia, Luís Marques Lito, José Melo-Cristino, Hermínia de Lencastre, Médicos Sentinela Network

**Affiliations:** 1 Laboratory of Molecular Genetics, Instituto de Tecnologia Química e Biológica (ITQB), Oeiras, Portugal; 2 Serviço de Patologia Clínica, Centro Hospitalar Lisboa Norte, Lisboa, Portugal; 3 Instituto de Microbiologia, Instituto de Medicina Molecular, Faculdade de Medicina, Universidade de Lisboa, Lisboa, Portugal; 4 Laboratory of Microbiology, The Rockefeller University, New York, United States of America; National Institutes of Health, United States of America

## Abstract

According to the EARS-Net surveillance data, Portugal has the highest prevalence of nosocomial methicillin-resistant *Staphylococcus aureus* (MRSA) in Europe, but the information on MRSA in the community is very scarce and the links between the hospital and community are not known. In this study we aimed to understand the events associated to the recent sharp increase in MRSA frequency in Portugal and to evaluate how this has shaped MRSA epidemiology in the community. With this purpose, 180 nosocomial MRSA isolates recovered from infection in two time periods and 14 MRSA isolates recovered from 89 samples of skin and soft tissue infections (SSTI) were analyzed by pulsed-field gel electrophoresis (PFGE), staphylococcal chromosome cassette *mec* (SCC*mec*) typing, *spa* typing and multilocus sequence typing (MLST). All isolates were also screened for the presence of Panton Valentine leukocidin (PVL) and arginine catabolic mobile element (ACME) by PCR. The results showed that ST22-IVh, accounting for 72% of the nosocomial isolates, was the major clone circulating in the hospital in 2010, having replaced two previous dominant clones in 1993, the Iberian (ST247-I) and Portuguese (ST239-III variant) clones. Moreover in 2010, three clones belonging to CC5 (ST105-II, ST125-IVc and ST5-IVc) accounted for 20% of the isolates and may represent the beginning of new waves of MRSA in this hospital. Interestingly, more than half of the MRSA isolates (8/14) causing SSTI in people attending healthcare centers in Portugal belonged to the most predominant clones found in the hospital, namely ST22-IVh (*n* = 4), ST5-IVc (*n* = 2) and ST105-II (*n* = 1). Other clones found included ST5-V (*n* = 6) and ST8-VI (*n* = 1). None of the MRSA isolates carried PVL and only five isolates (ST5-V-t179) carried ACME type II. The emergence and spread of EMRSA-15 may be associated to the observed increase in MRSA frequency in the hospital and the consequent spillover of MRSA into the community.

## Introduction

Methicillin-resistant *Staphylococcus aureus* (MRSA) is known to be a major cause of infections worldwide in the hospital and community settings, and while hospital-associated MRSA (HA-MRSA) generally affect patients with predisposing risk factors (prolonged hospitalization, use of indwelling catheters or prior surgical procedures) [1], community-associated MRSA (CA-MRSA) generally affect healthy and younger people without such risk factors [2]. Moreover, HA-MRSA and CA-MRSA belong to distinct genetic lineages, and while HA-MRSA are mostly multidrug-resistant and carry the larger SCC*mec* types I, II, III, CA-MRSA isolates frequently carry smaller SCC*mec* elements, usually type IV and V, and are resistant to fewer classes of antimicrobials [3]. Also, CA-MRSA isolates are strongly associated with virulence factors such as Panton-Valentine leukocidin (PVL) and the arginine catabolic mobile element (ACME) which are thought to contribute to their pathogenic potential [4], [5], [6].

Nowadays, however, CA-MRSA has been increasingly identified as a cause of hospital-onset and healthcare associated infections [7], [8]. On the other hand, hospital-associated clones have been described to cause infections in the community [9], [10] suggesting that certain clones have the ability to cross barriers between hospitals and the community.

In Portugal, MRSA prevalence is one of the highest in Europe, having sharply increased in the last decade from 31.9% in 2001 to 52.2% in 2010, which contrasts with the decreasing trend in MRSA prevalence observed for other countries such as England, France and Belgium [11], [12].

On the other hand and in spite of its clinical relevance, the prevalence and epidemiology of MRSA causing infection in the Portuguese community are not known. A single study assessing the contemporary causes of skin and soft tissue infections, which are the most common clinical manifestation of CA-MRSA, showed that Portugal, among all European countries, had the highest prevalence of MRSA causing SSTI between 1998 and 2004 [13].

Additionally, MRSA colonization in healthy young people was found to be extremely low (0.24% in 1996–1998 and 0.13% in 2006–2009) [14], [15]. Furthermore, only sporadic cases of CA-MRSA infections have been reported [16], [17] in Portugal.

In this study we aimed to understand the epidemiologic phenomenon associated to the recent sharp increase in MRSA frequency and to evaluate how this has shaped the MRSA population structure in the community. For this purpose we analyzed the clonal nature of MRSA during two time periods (1993 and 2010) spanning 17 years in a major tertiary teaching hospital in Portugal and compared it with the population structure of MRSA causing skin and soft tissue infections of people attending healthcare centers in the last time period (2010/2011). We hope to understand the extension of the epidemiological links between hospital and community in a country with a high prevalence of MRSA.

## Materials and Methods

### Ethical Statement

Isolates were obtained as part of routine diagnostic testing and were analyzed anonymously and the isolates, not humans, were studied. All data was collected in accordance with the European Parliament and Council decision for the epidemiological surveillance and control of communicable disease in the European community [18], [19].

### Study Design and Bacterial Collections

#### Hospital setting

Hospital de Santa Maria (HSM) is a 1,300-bed tertiary teaching hospital in Lisbon providing direct healthcare to approximately 350,000 people in Lisbon, but also providing services at a regional and national level.

Two collections were assembled at HSM in two time periods. Fifty-four MRSA isolates from inpatients were collected between January and March of 1993 from pus (50%), blood (22.2%), urine (9.3%), bronchial aspirates (7.4%), sputum (5.6%), catheter (3.7%) and pleural fluid (1.8%) [20]. A second collection of 520 MRSA isolates was recovered between January and December 2010, from which a subset of 180 isolates was selected according to the following criteria: the first fifteen isolates collected in each month, recovered from blood, pus and urine. From the 180 isolates collected in 2010 that were characterized in this study, 117 (65%) were from pus; 50 (27.8%) were from blood and 13 (7.2%) were from urine.

*S. aureus* were isolated at the hospital laboratory using standard procedures and identified as MRSA using the automated system VITEK2 *Advanced Expert System* (BioMérieux, Marcy, L’Étoile, France) or MicroScan® Walk-Away® (Siemens AG, Munich, Germany). All clinical and demographic data were recorded for each isolate.

#### Community setting

A total of 89 swabs from skin and soft tissue infections (SSTI) were recovered between November, 2010 and October, 2011 from people attending nine healthcare centers scattered throughout Portugal, mainly the north and center region (Águeda, Aveiro, Baltar, Braga, Cantanhede, Mealhada, Soure and Vila Real) and one located in the south (Lagos). For each sample, a questionnaire assessing risk factors for hospital contact was filled by the clinician. The questionnaires addressed the following questions: age and gender of the patient, previous contact with the hospital setting or surgery in the last 12 months, stay at a nursing home, previous colonization/infection with MRSA, presence of catheter or an indwelling device and previous antibiotic therapy in the last 12 months.

*S. aureus* were isolated by growth on mannitol salt agar and identification was performed by testing the production of clumping factor and protein A (Oxoid, Basingstoke, Hampshire, England). When the latex slide agglutination test produced inconclusive results, the production of coagulase was tested using the BD BBL Coagulase plasma, Rabbit test (Becton Dickinson, Sparks, Maryland, USA).

According to the information available from the questionnaires, isolates were considered to belong to two different groups as proposed by Salgado *et al.* [21]: community-onset MRSA (CO-MRSA) with healthcare-associated risk factors; and CO-MRSA without healthcare-associated risk factors.

#### Antibiotic susceptibility testing

All MRSA isolates were tested for antimicrobial susceptibility through disk diffusion method (Kirby-Bauer), according to the Clinical and Laboratory Standards Institute (CLSI) guidelines. Susceptibility was tested to a panel of 12 antibiotics: oxacillin, erythromycin, clindamycin, linezolid, ciprofloxacin, quinupristin-dalfopristin, sulfamethoxazole-trimethoprim, tetracycline, fusidic acid, rifampicin, vancomycin and gentamicin. In the case of fusidic acid, the European Committee on Antimicrobial Susceptibility Testing (EUCAST) guidelines from 2011 were used.

The minimal inhibition concentration (MIC) was determined by E-test (AB BioMérieux, Solna, Sweden) for isolates with a vancomycin inhibition halo ≤14 mm and for isolates susceptible to oxacillin by the disk diffusion method.

If isolates were resistant to three different antimicrobial classes, other than β-lactams, they were considered multidrug resistant.

### Molecular Characterization

#### Pulsed-field gel electrophoresis

All isolates were characterized by pulsed-field gel electrophoresis (PFGE) after digestion of the total DNA with SmaI, as described by Chung *et al*. [22]. PFGE patterns were analyzed with Bionumerics software (v6.6 Applied Maths, Saint-Martens-Latem, Belgium) with previously optimized parameters for *S. aureus* [23]. Dendrograms were constructed by the unweighted-pair group method using average linkages (UPGMA) and PFGE types and subtypes defined by groups formed at 80% and 95% Dice similarity cutoffs, respectively, as previously defined [23].

#### *mecA* detection and Staphylococcal Cassette Chromosome *mec* (SCC*mec*) typing

The presence of *mecA* gene was detected by PCR for all *S. aureus* isolates collected in the community setting as previously described [24]. SCC*mec* typing was directly performed by multiplex PCR [25] for all isolates from the hospital, since they had been previously identified as MRSA by the hospital clinical laboratory, and for all *S. aureus* isolates from the community that were positive for the presence of the *mecA* gene. The subtype of all isolates carrying SCC*mec* IV was identified by multiplex PCR as described before [26].

For those isolates in which SCC*mec* could not be assigned through the multiplex strategy, the *mec* gene complex class and *ccrAB*/*ccrC* were determined as previously described [27], [28], [29]. Confirmation of SCC*mec* V variant was achieved by long range PCR (Expand Long Template PCR System, Roche Diagnostics GmbH, Manheim, Germany) using the primers mecA P7 [24] and γR [29] to assure the linkage between *mec* complex C2 and *ccrC*.

#### *spa* typing

The *spa* typing was performed for at least one isolate from each different combination of PFGE type and SCC*mec* type. The *spa* polymorphic X region was amplified by PCR as described [30], [31] and the nucleotide sequence determined. The obtained sequences were analyzed using the Ridom StaphType program (v2.2.1, Ridom GmbH, Münster, Germany).

#### Multilocus sequence typing (MLST)

MLST was performed as previously described [32] on selected isolates (n = 23) representing each *spa* type present in both collections. The sequences were analyzed using the software SeqMan (v5.03, DNASTAR, Inc.) and submitted to the MLST database (http://saureus.mlst.net) for allele attribution. The eBURST algorithm (eBURST v.3) was applied to all MLST data in order to assign MLST clonal complexes (CC) (http://eburst.mlst.net). Last accessed at September 2, 2012.

#### Detection of Panton-Valentine leukocidin (PVL) and the arginine catabolic mobile element (ACME)

Panton-Valentine Leukocidin genes were detected by PCR as described by Lina *et al.* [33] and the presence and type of ACME was determined by the amplification of *arcA* [34] and *opp3* [5] genes as described before.

## Results

### Evolution of MRSA in a Single Hospital within a 17-year Period: Evidence of a Major Clonal Replacement

The molecular characterization of the 54 MRSA isolates recovered in the hospital in 1993 [20] showed that during this period, two clones were circulating in the hospital: the Iberian clone (ST247-I, t008/t051) and the Portuguese clone (ST239-III variant, t421), accounting for 72% (*n* = 39) and 28% (*n* = 15) of the isolates, respectively (see Table 1).

**Table 1:** Main characteristics of nosocomial MRSA isolates collected in 1993 and 2010.

Year	PFGE type, no. isolates (%)	SCC*mec* (no. isolates)	*spa* type^3^	ST (CC)^3^	Resistance Profile^4^ (no. isolates)
**1993**					
	M, 39 (72.2%)	I^1^ (39)	t008/t051	247 (8)	OX-ERY-CLI-CIP-TE-CN (8)
					OX-ERY-CIP-TE-RD-CN (8)
					OX-ERY-CIP-TE-CN (7)
					OX-ERY-CLI-CIP-TE-RD-CN (4)
					OX-ERY-CIP-QD-TE-CN (3)
					OX-ERY-CLI-CIP-QD-TE-RD-CN (1)
					OX-ERY-CLI-CIP-QD-TE-CN (1)
					OX-ERY-SXT-TE-CN (1)
					OX-ERY-CIP-QD-TE-RD-CN (1)
					OX-ERY-CIP-QD-TE-RD (1)
					OX-ERY-CIP-SXT-TE-CN (1)
					OX-ERY-CIP-SXT-TE (1)
					OX-ERY-CIP-TE-RD (1)
					OX-ERY-CIP-RD-CN (1)
	N, 12 (22.2%)	III variant^2^ (12)	t421	239 (8)	OX-ERY-CIP-QD-SXT-TE-CN (4)
					OX-ERY-SXT-TE-CN (2)
					OX-ERY-TE-RD-CN (2)
					OX-CIP-SXT-TE-CN (1)
					OX-ERY-CIP-SXT-TE (1)
					OX-ERY-CIP-QD-SXT-TE (1)
					OX-ERY-QD-SXT-TE-CN (1)
**2010**					
	O, 3 (5.6%)	III variant^2^ (3)	t421	239 (8)	OX-ERY-CIP-SXT-TE-CN (2)
					OX-ERY-CIP-TE-RD-CN (1)
	K, 129 (71.7%)	IVh (129)	t2357/t910/t025/t032/t1467/t1302	22 (22)	OX-ERY-CIP (94)
					OX-CIP (22)
					OX-ERY-CLI-CIP (4)
					OX-ERY-CIP-FD (3)
					OX-ERY-CLI-CIP-QD (2)
					OX-ERY-CIP-RD (1)
					OX-ERY-CIP-TE (1)
					OX-ERY-CIP-SXT (1)
					ERY-CIP (1)
	I, 38 (21.1%)	II (30)	t002	105 (5)	OX-ERY-CIP (16)
					OX-ERY-CLI-CIP (8)
					OX-ERY-CIP-FD (3)
					OX-ERY-CLI-CIP-FD (2)
					OX-ERY-CIP-RD (1)
		IVc (7)	t067/t002	125 (5)	OX-CIP (4)
					OX-ERY-CIP (2)
					OX-ERY-CLI-CIP-QD (1)
		IVc (1)	t535	5 (5)	OX-CIP-RD (1)
	F, 5 (2.8%)	III (3)	t037	2246 (8)	OX-ERY-CLI-CIP-SXT-TE-RD-CN (3)
		IVa (2)	t127	1 (15)	OX-ERY-TE (2)
	B, 4 (2.2%)	II (4)	t018	36 (30)	OX-ERY-CLI-CIP (4)
	C, 2 (1.1%)	IVc (1)	t008	8 (8)	OX-ERY-CIP (1)
		VI (1)	t024	8 (8)	OX-FD (1)
	E, 2 (1.1%)	II (2)	t002	105 (5)	OX-ERY-CIP (2)

1 One isolate with SCC*mec* type I variant: presence of *mec* complex class B and *ccrAB* type 1 was confirmed in simplex PCR;

2 No amplification could be retrieved from SCC*mec* type III J1 locus (primer RIF5 F10/RIF5 R13). The presence of *mec* complex class A and *ccrAB* type 3 was confirmed in simplex PCR;

3 *spa* typing and MLST were only performed on representative isolates of each PFGE type-SCC*mec* association;

4 OX, oxacillin; E, erythromycin; CLI, clindamycin; LZD, linezolid; CIP, ciprofloxacin; QD, quinupristin-dalfopristin; SXT, sulfamethoxazole-trimethoprim; TE, tetracycline; FD, fusidic acid; RD, rifampicin; VAN, vancomycin; CN, gentamicin.

Seventeen years later the MRSA population in this same hospital was completely different. From the 180 MRSA isolates collected in 2010 and selected for analysis, nine clonal types were identified. The major type found was ST22-IVh, the so-called EMRSA-15 clone, accounting for 72% of the isolates (*n* = 129). Six different *spa* types were identified among the 21 representative isolates of this clone tested: the t2357, t910, t025, t032, t1467 and t1302. The second most frequent clone was ST105-II-t002 (NY/Japan related) (18%, *n* = 30), followed by ST125-IVc-t067/t002 (Pediatric clone related) (4%, *n* = 7) and ST5-IVc-t535, the Pediatric clone, (a single isolate, 0.5%); all three clones belong to clonal complex 5 (CC5).

Together, the two major groups of isolates (EMRSA-15 and the group of the three different clones related to CC5) accounted for more than 90% of the isolates.

The remaining 6.2% of the isolates belonged to less prevalent clones such as ST2246-III-t037, ST8-IVc-t008 and ST8-VI-t024 (CC8), ST36-II-t018 (CC30) and ST1-IVa-t127 (CC15) (see Table 1). None of the MRSA isolates collected in the hospital during the two study periods carried PVL or ACME.

Concerning the antimicrobial resistance profiles, in 1993, all isolates were multidrug resistant, being resistant to oxacillin and at least four non β-lactam antibiotics. In contrast, in 2010, the majority exhibited a non-multidrug resistant pattern (81.7%), being mostly resistant to oxacillin (99%), ciprofloxacin (98%) and erythromycin (84%). One isolate belonging to ST22-IVh was susceptible to oxacillin with an MIC = 2 µg/ml.

### High Prevalence of Nosocomial MRSA Clones in the Community

A total of 89 swabs were collected from SSTI at the nine healthcare centers, from which 54 *S. aureus* isolates were isolated (60.7%). The mean age of the patients infected with *S. aureus* was 71 years old.

Among the 54 *S. aureus* isolates, 14 carried the *mecA* gene, which represents an MRSA infection rate of 25.9% among SSTI patients attending healthcare centers. However, of the 14 MRSA only two isolates were considered as CO-MRSA without risk factors. The other 12 isolates were collected from patients with at least one risk factor for hospital contact or did not have information in one or more questionnaire fields and were classified as CO-MRSA with risk factors.

The 14 MRSA isolates belonged to five different clones: ST5-V (*n* = 6) associated to *spa* types t179 and t442; ST22-IVh (EMRSA-15 clone) (*n* = 4) associated to *spa* types t2357, t032 and t5888; ST5-IVc (Pediatric clone) (*n* = 2) characterized by *spa* type t535; ST105-II (New York/Japan clone related) (*n* = 1) characterized by *spa* type t002 and ST8-VI (*n* = 1) characterized by *spa* type t008 (see Table 2). Interestingly, most of the isolates belong to clones that were also found in the hospital setting. Three isolates belonging to ST5-V-t179, carried additionally to SCC*mec* type V, a *ccrAB1* that might correspond to the presence of an additional SCC element. In these isolates the presence of SCC*mec* V, and thus the linkage of *mec* complex C2 to *ccrC* and not to *ccrAB1*, was confirmed by long-range PCR.

**Table 2:** Main characteristics of the 14 MRSA isolates collected in the healthcare centers in 2010/2011.

PFGE type, no.isolates (%)	SCC*mec*(no. isolates)	*spa* type^2^(no. isolates)	ST (CC)^2^	PVL	ACME(no. isolates)	Resistance Profile^3^(no. isolates)
I, 6 (43%)	IVc (2)	t535	5 (5)	–	–	CIP-FD (1)
						OX-ERY-CIP-QD-FD (1)
	V (3)	t179	5 (5)	–	*arcA*+(2)	CIP-CN (2)
		t442	5 (5)	–	–	CIP-SXT-TE-CN (1)
	II (1)	t002	105 (5)	–	–	OX-ERY-CIP (1)
P, 3 (21%)	V^1^ (3)	t179	5 (5)	–	*arcA*+(3)	CIP-FD-CN (1)
						ERY-CIP-FD-CN (1)
						ERY-CIP-QD-FD-CN (1)
K, 4 (29%)	IVh (4)	t032/t5888/t2357	22 (22)	–	–	OX-ERY-CIP (2)
						OX-CIP (1)
						OX-ERY-CLI-CIP-QD (1)
Q, 1 (7%)	VI (1)	t008	8 (8)	–	–	CIP-FD (1)

1 Isolates carry the *ccrAB1*, *ccrC* and *mec* complex C2. SCC*mec* type was confirmed by long-range PCR;

2 *spa* typing and MLST were only performed on representative isolates of each PFGE type-SCC*mec* association.

3 OX, oxacillin; E, erythromycin; CLI, clindamycin; LZD, linezolid; CIP, ciprofloxacin; QD, quinupristin-dalfopristin; SXT, sulfamethoxazole-trimethoprim; TE, tetracycline; FD, fusidic acid; RD, rifampicin; VAN, vancomycin; CN, gentamicin.

None of the MRSA isolates carried PVL; however, five isolates (36%) belonging to ST5-V-t179, carried ACME type II with the amplification of the *arcA* gene but not the *opp3* gene and were all collected from the same health-care center in the south of Portugal.

Considering antimicrobial resistance profiles, all isolates were resistant to ciprofloxacin, 50% to erythromycin and 43% to gentamicin. Of note, eight isolates (belonging to ST5-V and ST8-VI) were susceptible to oxacillin with an MIC ≤2 µg/ml, in spite of the fact that they carried *mecA*.

Furthermore, six out of the 14 MRSA isolates exhibited a multidrug resistance profile, being resistant to three or more classes of antibiotics other than β-lactams (see Table 2).

### Epidemiological Links between the Hospital and the Community Settings

Comparing the populations of MRSA recovered in the community and hospital in 2010, it can be observed that some isolates from both settings share exactly the same molecular characteristics, namely PFGE type, SCC*mec* type, *spa* type and ST. This was the case of isolates belonging to ST22-IVh-t2357, ST22-IVh-t032, ST105-II-t002 and ST5-IVc-t535 that had the same genetic features independently of the fact that they were isolated in the hospital or in the community. In addition, five of these isolates showed an identical antimicrobial resistance profile.

**Figure 1:**
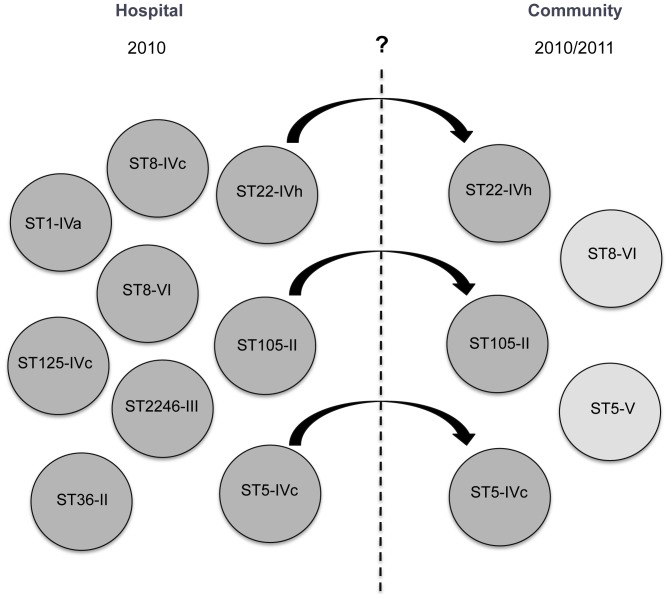
MRSA population structure in the hospital and community in Portugal. Schematic representation of the MRSA population structure in the hospital and community settings and the possible dissemination of hospital clones into the community.

Overall, our results suggest the occurrence of dissemination of typical HA-MRSA clones into the community (see Figure 1).

## Discussion

In a major tertiary teaching hospital in Portugal, the frequency of MRSA has sharply increased from 30% in 1993 to 49% in 2010. This is a picture that mirrors the situation observed in most of the hospitals in Portugal (Faria *et al.*, unpublished). However, the reasons lying behind this high prevalence are complex and were not explored in this study.

Our results showed that this steep increase in MRSA frequency was associated with a major clonal shift, in which the Portuguese and Iberian clones were replaced by EMRSA-15 and some less prevalent clones.

In fact, no common PFGE types, *spa* types (except for t008) or STs were found between the two study periods. The only CC that prevailed between the two study periods was CC8, although associated to distinct clones (the ST247-I and ST239-III variant in 1993 and ST2246-III and ST8-IV/VI in 2010). Moreover, a major change in the antibiogram profile was concomitantly observed between the two periods. Whereas in 1993 isolates were resistant to almost all antimicrobial agents tested, in 2010 the great majority of isolates was non-multidrug resistant (81.7%).

The establishment of the non-multidrug resistant clone ST22-IVh, or EMRSA-15, as the major clone in hospitals in Portugal, was previously reported [35], [36], suggesting that this clone has been in Portugal for at least 11 years. EMRSA-15 was first described in the United Kingdom, where it became the major HA-MRSA clone, together with EMRSA-16, accounting for more than 95% of bacteremia in this country [37]. It has also spread throughout Europe, being reported in Germany, Malta, Italy [38], [39], [40], and representing the major clone in hospitals in Majorca and the Czech Republic [41], [42].

The clonal replacement that occurred within the major hospital analyzed in this study, has occurred in the past in Portugal [35], and also in other parts of the world [38], [39], [43], [44], [45], [46]. Although the reasons lying behind the fade of one clone and the emergence of another are not known, it is believed that the newly introduced clone has a higher fitness than the displaced one.

EMRSA-15 carries few resistant traits, the small SCC*mec* type IV and has an increased capacity to form biofilm [47]. Moreover, its capacity of dissemination and invasion can be enhanced by the acquisition of additional virulence factors like ACME and PVL [48], [49]. Altogether, these characteristics may have conferred to this particular clone a higher epidemicity, growth rate and capacity for persistence that could explain its success and fitness and may also explain the increase of the MRSA frequency observed in the hospital between 1993 and 2010.

Besides EMRSA-15, we observed that two additional clones (ST105-II and ST125-IVc), belonging to CC5, accounted for a significant part of the MRSA population (22%). Previously, the New York/Japan clone was observed in the nosocomial setting in Portugal and had been suggested as the most probable next emergent MRSA clone in the nosocomial setting in this country [35]. However, this role appears to have been taken by its descendants, the ST105-II and ST125-IVc. These clones differ from ST5 by one point mutation [50] on *yqil* locus and have already been described as major clones in hospitals in Switzerland [43] and Spain [51] and as minor clones in Brazil [52], the USA [53] and Norway [54]. However, as far as we are concerned, this is the first time that ST105-II and ST125-IVc are reported in a Portuguese hospital. Since these clones have already proven to be capable of disseminating in the hospital and becoming dominant, it is worth watching for the replacement of EMRSA-15 by ST105-II and/or ST125-IVc.

Besides, being one of the countries with the highest prevalence of MRSA in hospitals, Portugal is also the European country with the highest prevalence of MRSA causing skin and soft tissue infections, the most common manifestation of community-associated MRSA disease [13]. In the study presented here, we documented for the first time the characterization of MRSA causing SSTI in persons attending healthcare centers in Portugal and verified that the rate of MRSA causing SSTI (≈26%) in this population was alarmingly high.

However, this study has some limitations since we cannot ignore the fact that a low number of samples were recovered, which could have influenced the MRSA rate obtained. Also, the type of population analyzed may have not been the most adequate to evaluate the prevalence of CA-MRSA since it was composed in its majority of old people (median age 71) with risk factors for hospital contact. The rate of MRSA in SSTI is much higher than that found in nasopharyngeal colonization in children in Portugal [15], but similar to the rates of MRSA in long-term care facilities (Miragaia *et al*., unpublished), suggesting that probably elderly, rather than children, may constitute a reservoir of MRSA in the community in Portugal.

Almost half of the MRSA isolates identified as causing SSTI in the community belonged to clone ST5-V, associated to ACME II. This clonal type was recently described as a possible emerging CA-MRSA clone in Japan [55], and might be emerging in Portugal as well.

We did not find representatives of the USA300 and ST80-IV clones, which are typical pandemic CA-MRSA clones. This clearly contrasts with the situation observed in Europe and the USA, wherein nowadays these are the most frequent clones in the community [56], [57]. This, however, may not be surprising since the prevalence of isolates belonging to epidemic CA-MRSA clones in Portugal continues to be low (<3%) (Tavares *et al*. unpublished).

Interestingly, the other half of MRSA found causing SSTI belonged to the major HA-MRSA clones found in the hospital setting in this study (ST22-IVh-t032/t2357, ST105-II-t002 and ST5-IVc-t535) sharing the same PFGE, *spa* type, SCC*mec* and ST. Moreover, five isolates shared the same antibiotic susceptibility profile. Altogether, these results suggest a spillover of HA-MRSA isolates into the community.

Of notice, all MRSA recovered from SSTI in this study were resistant to ciprofloxacin, which might reflect the trend of antibiotic consumption in the outpatient setting in Portugal, since quinolones are the third most prescribed antimicrobials [58] and, accordingly, ciprofloxacin (a fluoroquinolone) was prescribed to at least 50% of the patients with MRSA infection in our study (data not shown).

Also of interest was the fact that eight MRSA isolates belonging to ST5-V (CC5) and ST8-VI (CC8) were susceptible to oxacillin, in spite of the fact that they carried *mecA*. Sharff, K.A. *et al* [59], recently described two cases of infections caused by oxacillin susceptible MRSA strains belonging to these exact same clonal complexes, one of which was fatal. Since oxacillin-susceptible MRSA strains from this study carry SCC*mec* V and VI, which lack functional *mecA* regulators, the failure to detect the expression of beta-lactams resistance *in vitro* probably results from the fact that they have a heterogeneous profile of resistance, rather than from the lack of induction by beta-lactams. In hospitals MRSA identification relies on antimicrobial susceptibility testing only, which in cases like this, can lead to treatment failure and increased burden of disease.

The vehicles of dissemination of HA-MRSA from the hospital into the community are not firmly established and may differ in different countries. A recent study showed that public buses could function as an important reservoir of this clone in the community [60]. Nevertheless, patients themselves and healthcare workers may be the main means of dispersal. In fact, a great part of MRSA collected from SSTI were isolated from elderly people, whom for their intrinsic condition have a higher probability of having a history of hospitalization and surgery or other risk factors associated with a previous contact with healthcare-associated MRSA, and could have served as a means of spreading of MRSA from the hospital in the community.

The spillover of *S. aureus* from the hospital into the community observed in this study probably results from the high frequencies of MRSA observed inside the hospital setting, like was observed before for penicillin resistant *S. aureus* [61]. Nowadays, penicillin resistant *S. aureus* are endemic in the community, and is worrisome to assume that a similar situation might occur with nosocomial multidrug resistant MRSA in the future in Portugal.

Overall, our findings showed that the increase in the rate of MRSA in a major hospital in Portugal was associated to a clonal replacement and to the emergence of EMRSA-15. Moreover, we showed that in a country with a high rate of nosocomial MRSA, the population structure of MRSA in the community might mirror that found in the hospital setting.
